# Common Variants on Cytotoxic T Lymphocyte Antigen-4 Polymorphisms Contributes to Type 1 Diabetes Susceptibility: Evidence Based on 58 Studies

**DOI:** 10.1371/journal.pone.0085982

**Published:** 2014-01-23

**Authors:** Jingnan Wang, Lianyong Liu, Junhua Ma, Fei Sun, Zefei Zhao, Mingjun Gu

**Affiliations:** Department of Endocrine, Shanghai Pudong New Area Gongli Hospital, Shanghai, People's Republic of China; Children's Hospital Boston, United States of America

## Abstract

In the past decade, a number of case–control studies have been carried out to investigate the relationship between the *CTLA4* gene polymorphisms and type 1 diabetes (T1D). However, these studies have yielded contradictory results. To investigate this inconsistency, we performed a meta-analysis of all available studies dealing with the relationship between the *CTLA4* polymorphism and T1D. In total, 58 association studies on two *CTLA4* polymorphisms (G49A and C60T) and risk of T1D, including a total of 30,723 T1D cases and 45,254 controls were included. In a combined analysis, the summary per-allele odds ratio (OR) for T1D of the G49A and C60T polymorphism was 1.42 [95% confidence interval (CI): 1.31–1.53, P<10^−5^] and 1.23 (95% CI: 1.18–1.29, P<10^−5^), respectively. Significant results were also observed using dominant or recessive genetic model. In the subgroup analysis by ethnicity and sample size, significantly increased risks were also found for these polymorphisms. This meta-analysis demonstrated that the G49A and C60T polymorphism of *CTLA4* is a risk factor associated with increased T1D susceptibility, but these associations vary in different ethnic populations.

## Introduction

Type 1 diabetes (T1D) is an autoimmune disease characterized by destruction of the insulin-producing β-cells in the pancreatic islets. Although its etiology is not yet understood, strong genetic and environmental components appear to modulate individual disease susceptibility in patients and in animal models [1]. The cytotoxic T lymphocyte antigen-4 gene (*CTLA4*) and the gene encoding CD28 have been mapped to chromosome 2q33. CTLA4 is a glycoprotein receptor expressed on activated T cells and CD28 is involved in the regulation process of the activation of T cells by antigen-presenting cells and subsequent cellular immunity [2]. Based on its role in the regulation of the activation of T cells and T cell and B cell interactions [3], *CTLA4* has been considered to be a permissive candidate gene involved in the etiology of autoimmune diseases. A number of common polymorphisms have been reported both in the coding and promoter regions of the *CTLA4* gene. Among them, one common polymorphism in the coding region, which leads to a alanine→threonine substitution at exon 1 (G49A, rs231775) and one located at 3′-UTR (G6230A, C60T, rs3087243) were studied widely for their association with T1D susceptibility.

In the past decade, several association studies have investigated the associations between the *CTLA4* gene and T1D susceptibility. However, these studies yielded conflicting results. Genetic association studies can be problematic to reproduce due to inadequate statistical power, multiple hypothesis testing, population stratification, publication bias, and phenotypic heterogeneity. In addition, with the increased studies in recent years among Caucasian, Asian, and other populations, there is a need to reconcile these data. Therefore, we performed a systematic meta-analysis of published studies to clarify the relationship between *CTLA4* and T1D.

## Materials and Methods

### Literature search strategy and inclusion criteria

The literature included in our analysis was selected from PubMed, EMBASE, ISI web of science and Chinese National Knowledge Infrastructure with keywords relating to the relevant genes (e.g. ‘cytotoxic T lymphocyte antigen-4’ or ‘*CTLA4*’) in combination with words related to T1D (e.g. ‘Type 1 diabetes’ or ‘insulin dependent diabetes mellitus’) and ‘polymorphism’ or ‘variation’. Genetic association studies published before the 31 Jan. 2013 on T1D and polymorphisms in the *CTLA4* gene described above were retrieved, and their references were checked to identify other relevant publications. The search was supplemented by reviews of reference lists for all relevant studies and review articles. The major inclusion criteria were (a) original papers containing independent data, (b) case–control or cohort studies and (c) available genotype distribution information or odds ratio (OR) with its 95% confidence interval (OR) with its 95% confidence interval (CI) and P-value. The major reasons for exclusion of studies were (a) overlapping data and (b) case-only studies and review articles.

### Data extraction

Data extraction was performed independently by two reviewers, and differences were resolved by further discussion among all authors. For each included study, the following information was extracted according to a fixed protocol: first author's surname, publication year, definition and numbers of cases and controls, diagnostic criterion, frequency of genotypes, source of controls, gender, age at onset, Hardy–Weinberg equilibrium (HWE) status, ethnicity and genotyping method.

### Statistical methods

The strength of association between polymorphisms of *CTLA4* and T1D risk was assessed by OR with the corresponding 95% CI. The per-allele OR of the risk allele was compared between cases and controls. Then, we examined the association between risk genotype of polymorphisms and T1D susceptibility using dominant and recessive genetic models.

Heterogeneity across individual studies was calculated using the Cochran chi-square Q test followed by subsidiary analysis or by random-effects regression models with restricted maximum likelihood estimation [4]–[6]. Random-effects and fixed-effect summary measures were calculated as inverse variance-weighted average of the log OR. The results of random-effects summary were reported in the text because they take into account the variation between studies. In addition, we investigated potential sources of identified heterogeneity among studies by stratifying by ethnic group and the number of cases (≥300 and <300). Ethnic group was defined as East Asians, Caucasians (i.e. people of European origin) and Middle Eastern (e.g. Iran, Egyptian and Lebanon), Indian and African. The Z test was used to determine the significance of the pooled OR.

We assessed publication bias by using an ancillary procedure attributed to Egger et al. [7], which uses a linear regression approach to measure funnel plot asymmetry on the natural logarithm of the OR. The larger the deviation from the funnel curve of each study, the more pronounced the asymmetry will be. The results from small studies tend to scatter widely at the bottom of the graph, with the spread narrowing among larger studies. The significance of the intercept is evaluated using the t test. Sensitivity analysis was performed by removing each individual study in turn from the total and re-analyzing the remainder. This procedure was used to ensure that no individual study was entirely responsible for the combined results. All statistical analyses were carried out with the Stata software version 10.0 (Stata Corporation, College Station, TX, USA). The type I error rate was set at 0.05. All the P-values were for two-sided analysis.

## Results

### Characteristics of included studies

The combined search yielded 193 references. 135 articles were excluded because they did not meet the criteria or reported overlapping data (Figure S1). Finally, a total of 58 case–control studies were retrieved based on the search criteria for T1D susceptibility related to the *CTLA4* polymorphisms [8]–[65]. The main study characteristics were summarized in Table 1. There are 51 studies with 10,969 T1D cases and 14,111 controls concerning G49A polymorphism and 15 studies with 22,437 T1D cases and 34,599 controls concerning C60T variation. These two polymorphisms were found to occur in frequencies consistent with HWE in the control populations of the vast majority of the published studies.

**Table 1:** Characteristics of the studies included in the meta-analysis.

Study	Year	Ethnicity	Case	Control	No. of case/control	Genotyping method	Mean age at onset
Nistico [8]	1996	Belgian	T1D per NDDG criteria	Non-diabetic participants	483/529	allele-specific PCR	NA
Donner [9]	1997	American	T1D patients	Healthy	293/325	SSCP	17.9
Van der Auwera [10]	1997	Belgian	T1D per NDDG criteria	Healthy	425/530	RFLP	20.0
Awata [11]	1998	Japanese	T1D patients	Healthy	173/425	NA	24.5
Djilali-Saiah [12]	1998	French	T1D patients	Healthy	112/100	NA	24.9
Krokowski [13]	1998	Polish	T1D patients	Healthy	192/136	allele-specific PCR	9.5
Abe [14]	1999	Japanese	T1D per NDDG criteria	Healthy	111/445	RFLP	NA
Hayashi [15]	1999	Japanese	T1D per ADA criteria	Healthy	117/141	RFLP	34.0
Yanagawa [16]	1999	Japanese	T1D patients	Non-diabetic participants	110/200	RFLP	25.9
Lee [17]	2000	Chinese	T1D per NDDG criteria	Non-diabetic participants	253/91	RFLP	7.1
Takara [18]	2000	Japanese	T1D patients	Healthy	74/107	RFLP	21.8
Ihara [19]	2001	Japanese	T1D per NDDG criteria	Non-diabetic participants	160/200	SSCP	7.9
Kamoun Abid [20]	2001	Tunisian	T1D patients	Healthy	74/49	RFLP	10.3
Kikuoka [21]	2001	Japanese	T1D per WHO criteria	Non-diabetic participants	125/200	RFLP	NA
McCormack [22]	2001	Irish	T1D patients	Healthy	130/307	NA	NA
Osei-Hyiaman [23]	2001	Chinese, African	T1D per NDDG criteria	Healthy	532/621	SSCP	NA
Cinek [24]	2002	Czech	T1D per WHO criteria	Non-diabetic participants	305/289	allele-specific PCR	7.6
Cosentino [25]	2002	Italian	T1D patients	Healthy	80/85	RFLP	NA
Fajardy [26]	2002	French	T1D per WHO criteria	Non-diabetic participants	134/273	RFLP	17.0
Klitz [27]	2002	Philippine	T1D per ADA criteria	Non-diabetic participants	90/94	allele-specific PCR	NA
Ma [28]	2002	Chinese	T1D per ADA criteria	Healthy	31/36	RFLP	NA
Ongagna [29]	2002	French	T1D per WHO criteria	Non-diabetic participants	62/84	RFLP	13.3
Wood [30]	2002	German	T1D patients	Non-diabetic participants	176/220	RFLP	NA
Bouqbis [31]	2003	Moroccan	T1D patients	Healthy	118/114	SNaPshot	NA
Mochizuki [32]	2003	Japanese	T1D per ADA criteria	Non-diabetic participants	97/60	RFLP	NA
Haller [33]	2004	Estonian	T1D per ECDC criteria	Healthy	69/158	RFLP	NA
Ide [34]	2004	Japanese	T1D per ADA criteria	Healthy	116/114	RFLP	22.0
Liang [35]	2004	Japanese	T1D per ADA criteria	Normal glucose tolerance	29/40	RFLP	25.3
Zalloua [36]	2004	Lebanese	T1D patients	Healthy	190/96	allele-specific PCR	8.9
Caputo [37]	2005	Argentinean	T1D per WHO criteria	Healthy	123/168	RFLP	15.0
Mojtahedi [38]	2005	Iranian	T1D per NDDG criteria	Healthy	109/331	SSCP	16.4
Zhernakova [39]	2005	Dutch	IS-PAD	Healthy	350/900	TaqMan	17.0
Ahmedov [40]	2006	Azeri	T1D per WHO criteria	Non-diabetic participants	160/271	SSCP	9.1
Baniasadi [41]	2006	Indian	T1D per ADA criteria	Healthy	130/180	RFLP	15.4
Kanazawa [42]	2006	Japanese	T1D patients	Normal glucose tolerance	71/39	RFLP	35.4
Ikegami [43]	2006	Japanese	T1D patients	Non-diabetic participants	769/723	Invader	27.3
Haller [44]	2007	Estonian	T1D per ECDCD criteria	Healthy	70/252	RFLP	24.3
Howson [45]	2007	British	T1D patients	Non-diabetic participants	4066/6866	TaqMan	7.5
Butty [46]	2008	American	T1D patients	Normoglycemic participants	224/343	TaqMan	NA
Kawasaki [47]	2008	Japanese	T1D per WHO criteria	Healthy	91/369	RFLP	NA
Saleh [48]	2008	Egyptian	T1D patients	Healthy	396/396	SSCP	6.7
Smyth [49]	2008	British	T1D patients	Healthy	5253/9161	TaqMan	7.5
Balic [50]	2009	Chilean	T1D per ADA criteria	Healthy	300/310	RFLP	8.9
Douroudis [51]	2009	Estonian, Finnish	T1D per WHO criteria	Healthy	574/955	TaqMan	NA
Jin [52]	2009	Chinese	T1D per WHO criteria	Healthy	413/476	RFLP	17.0
Jung [53]	2009	Korean	T1D per WHO criteria	Healthy	176/90	RFLP	7.5
Korolija [54]	2009	Croatian	T1D patients	Healthy	102/193	RFLP	11.5
Lemos [55]	2009	Portuguese	T1D patients	Healthy	207/249	RFLP	16.1
Momin [56]	2009	Chilean	T1D per ADA criteria	Healthy	261/280	RFLP	8.2
Benmansour [57]	2010	Tunisian	T1D patients	Normal glucose tolerance	228/193	RFLP	15.7
Klinker [58]	2010	Finnish	T1D patients	Normoglycemic participants	591/1538	TaqMan	26.0
Howson [59]	2011	British	T1D per WHO criteria	Normoglycemic participants	928/2043	TaqMan	33.3
Philip [60]	2011	Indian	T1D patients	Healthy	53/53	RFLP	NA
Plagnol [61]	2011	British	T1D patients	Healthy	8506/10596	Affymetrix chip	8.0
Reddy [62]	2011	American	T1D per ADA criteria	Healthy	1434/1864	TaqMan	NA
Wafai [63]	2011	Lebanese	T1D per ADA criteria	Healthy	39/46	RFLP	8.9
Horie [64]	2012	Japanese	T1D patients	Normoglycemic participants	134/222	RFLP	NA
Mosaad [65]	2012	Egyptian	T1D per ADA criteria	Healthy	104/78	RFLP	8.2

NA: Not Available, WHO: World Health Organization, ADA: American Diabetes Association, ECDC: Expert Committee on the Diagnosis and Classification of Diabetes Mellitus, IS-PAD: International Society of Paediatric and Adolescent Diabetes.

### Association of CTLA4 G49A polymorphism and T1D

**Figure 1:**
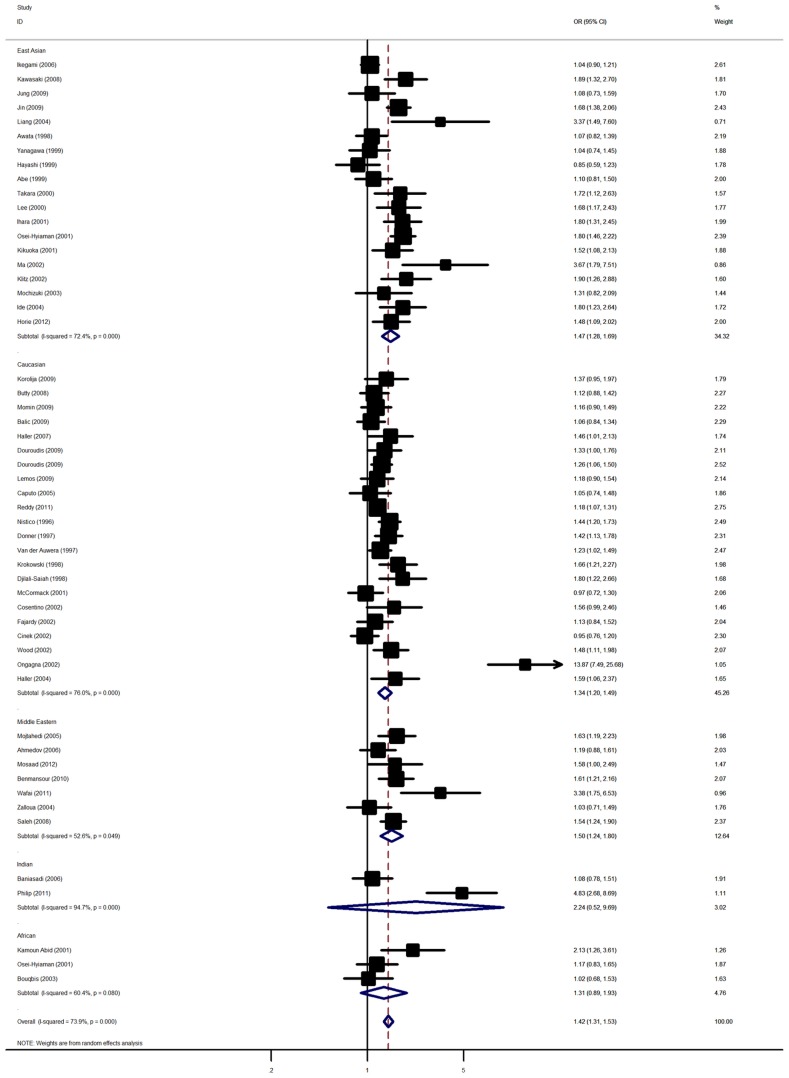
Forest plot from the meta-analysis of type 1 diabetes risk and *CTLA4* G49A polymorphism.

Overall, there was evidence of an association between the increased risk of T1D and the variant in different genetic models when all the eligible studies were pooled into the meta-analysis. Using random effect model, the summary per-allele OR of the G variant for T1D was 1.42 [95% CI: 1.31–1.53; *P*(Z)<10^−5^; *P*(Q)<10^−5^; Figure 1], with corresponding results under dominant and recessive genetic models of 1.48 [95% CI: 1.31–1.66; *P*(Z)<10^−5^; *P*(Q)<10^−5^ ] and 1.68 [95% CI: 1.47–1.91; *P*(Z)<10^−5^; *P*(Q)<10^−5^], respectively.

In the stratified analysis by ethnicity, significantly increased risks were found among East Asian populations [G allele: OR = 1.47, 95% CI: 1.28–1.69; dominant model: OR = 1.65, 95% CI: 1.29–2.11; recessive model: OR = 1.65, 95% CI: 1.35–2.02] and Caucasian populations [G allele: OR = 1.23, 95% CI: 1.20–1.49; dominant model: OR = 1.31, 95% CI: 1.14–1.49; recessive model: OR = 1.68, 95% CI: 1.37–2.06]. Similar significant associations were also observed for Middle Eastern population [G allele: OR = 1.50, 95% CI: 1.24–1.80; dominant model: OR = 1.62, 95% CI: 1.15–2.29; recessive model: OR = 1.93, 95% CI: 1.26–2.96]. However, no significant associations were detected among Indian and African populations (Table 2). Subsidiary analyses of sample size yielded a per-allele OR for small studies of 1.52 (95% CI: 1.34–1.72) and for large studies of 1.30 (95% CI: 1.19–1.42).

**Table 2:** Meta-analysis of the *CTLA-4* G49A polymorphism on type 1 diabetes risk.

Sub-group analysis	No. of cases/controls	G allele vs. A allele			Dominant model			Recessive model		
		OR (95%CI)	P(Z)	P(Q)	OR (95%CI)	P(Z)	P(Q)	OR (95%CI)	P(Z)	P(Q)
Total	10969/14111	1.42 (1.31–1.53)	<10^−5^	<10^−5^	1.48 (1.31–1.66)	<10^−5^	<10^−5^	1.68 (1.47–1.91)	<10^−5^	<10^−5^
Ethnicity										
East Asians	3430/4453	1.47 (1.28–1.69)	<10^−5^	<10^−5^	1.65 (1.29–2.11)	<10^−4^	0.008	1.66 (1.35–2.02)	<10^−5^	0.0009
Caucasians	5756/7650	1.23 (1.20–1.49)	<10^−5^	<10^−5^	1.31 (1.14–1.49)	<10^−4^	0.002	1.68 (1.37–2.06)	<10^−5^	<10^−5^
Middle Eastern	1226/1411	1.50 (1.24–1.80)	<10^−4^	0.05	1.62 (1.15–2.29)	0.006	0.0007	1.93 (1.26–2.96)	0.003	0.07
African	374/364	1.31 (0.89–1.93)	0.17	0.001	1.43 (0.84–2.44)	0.18	0.11	1.31 (0.61–2.82)	0.49	0.12
Indian	183/233	2.24 (0.52–9.69)	0.28	0.08	3.94 (0.33–47.54)	0.28	<10^−4^	1.89 (0.48–7.41)	0.36	0.02
Sample size										
Small	4224/6368	1.52 (1.34–1.72)	<10^−5^	<10^−4^	1.58 (1.32–1.88)	<10^−5^	<10^−5^	1.77 (1.46–2.16)	<10^−5^	<10^−5^
Large	6745/7743	1.30 (1.19–1.42)	<10^−5^	<10^−5^	1.39 (1.20–1.61)	<10^−4^	0.001	1.58 (1.38–1.82)	<10^−5^	0.09

### Association of CTLA4 C60T polymorphism and T1D

**Figure 2:**
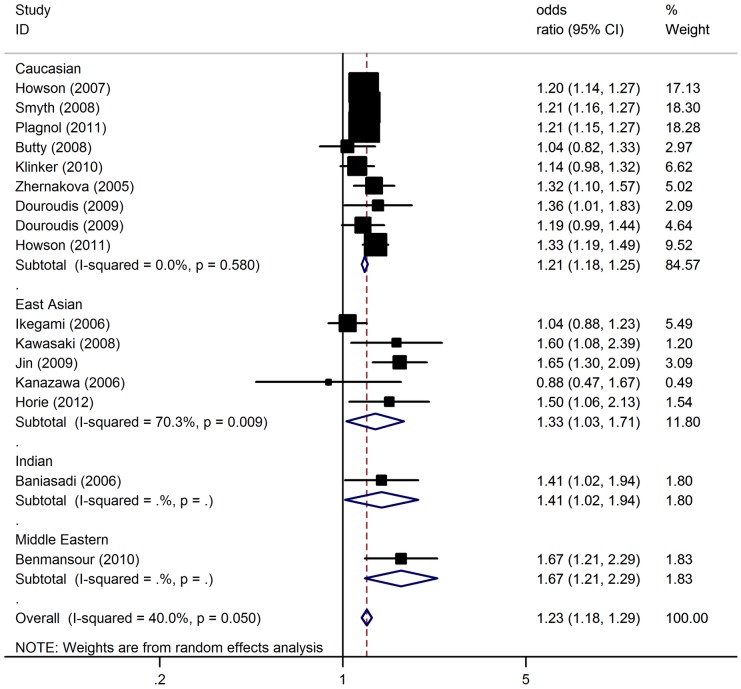
Forest plot from the meta-analysis of type 1 diabetes risk and *CTLA4* C60T polymorphism.

In the overall analysis, the C60T polymorphism of *CTLA4* was significantly associated with elevated T1D risk with a per-allele OR of 1.23 [95% CI: 1.18–1.29; *P*(Z)<10^−5^; *P*(Q) = 0.05; Figure 2]. Significant associations were also found under dominant [OR = 1.31; 95% CI: 1.16–1.47; *P*(Z)<10^−5^; *P*(Q) = 0.11] and recessive [OR = 1.32; 95% CI: 1.19–1.43; *P*(Z)<10^−5^; *P*(Q) = 0.06] genetic model.

When studies were stratified for ethnicity, significant risks were found among Caucasians in all genetic models [C allele: OR = 1.21, 95% CI: 1.18–1.25; dominant model: OR = 1.31, 95% CI: 1.20–1.44; recessive model: OR = 1.24, 95% CI: 1.18–1.31]. Similar results were also found in the Middle Eastern populations and Indians with a per-allele OR of 1.67 (95% CI: 1.21–2.29) and 1.41 (95% CI: 1.02–1.94), respectively. Only marginal significant results were detected for East Asians with per-allele OR of 1.33 (95% CI: 1.03–1.71). In the stratified analysis by sample size, significant associations were detected in both large and small studies (Table 3).

**Table 3:** Meta-analysis of the *CTLA-4* C60T polymorphism on type 1 diabetes risk.

Sub-group analysis	No. of cases/controls	C allele vs. T allele			Dominant model			Recessive model		
		OR (95%CI)	P(Z)	P(Q)	OR (95%CI)	P(Z)	P(Q)	OR (95%CI)	P(Z)	P(Q)
Total	22437/34599	1.23 (1.18–1.29)	<10^−5^	0.05	1.31 (1.16–1.47)	<10^−5^	0.11	1.32 (1.19–1.43)	<10^−5^	0.06
Ethnicity										
East Asians	1487/1821	1.33 (1.03–1.71)	0.03	0.009	1.18 (0.58–2.43)	0.65	0.06	1.44 (1.00–2.03)	0.05	0.02
Caucasians	20592/32405	1.21 (1.18–1.25)	<10^−5^	0.58	1.31 (1.20–1.44)	<10^−5^	0.26	1.24 (1.18–1.31)	<10^−5^	0.43
Middle Eastern	228/193	1.67 (1.21–2.29)	0.002	NA	1.63 (1.10–2.42)	0.01	NA	2.37 (1.18–4.76)	0.01	NA
Indian	130/180	1.41 (1.02–1.94)	0.04	NA	1.74 (1.06–2.87)	0.03	NA	1.34 (0.78–2.33)	0.29	NA
Sample size										
Small	2226/3680	1.35 (1.21–1.50)	<10^−5^	0.15	1.39 (1.04–1.86)	0.02	0.09	1.51 (1.30–1.75)	<10^−5^	0.34
Large	20211/30919	1.21 (1.17–1.25)	<10^−5^	0.25	1.34 (1.23–1.46)	<10^−5^	0.21	1.22 (1.16–1.29)	<10^−5^	0.47

NA: not available.

### Haplotype analysis

The linkage disequilibrium (LD) analysis revealed a tight LD between the G49A and C60T sites with D′ score of 0.95. Haplotype analyses between 49G>A, and C60T polymorphisms were performed in the 4 studies, involving 2242 cases and 2581 controls. Three prevalent haplotypes (Table S1), which represent more than 95% of the haplotypes among those studied at the CTLA4 loci (49G>A, C60T), were detected both in affected and unaffected subjects. The AT haplotype was significantly associated with decreased diabetes risk in the overall analysis (OR = 0.87, 95% CI: 0.77–0.98, P = 0.03). In addition, the frequency of GC haplotype (OR = 1.12, 95% CI: 0.98–1.28, P = 0.10) was also higher in diabetes patients compared with controls.

### Sensitivity analyses and publication bias

Sensitivity analysis indicated that no single study influenced the pooled OR qualitatively, suggesting that the results of this meta-analysis are stable (data not shown). A funnel plot of these included studies suggested a possibility of the preferential publication of positive findings in smaller studies for G49A polymorphism of *CTLA4* (Begg test, P = 0.001; Egger test, P = 0.0002; Figure S2). The Duval and Tweedie nonparametric “trim and fill” method was used to adjust for publication bias. Meta-analysis with and without “trim and fill” method did not draw different conclusion (data not shown), indicating that our results were statistically robust. The shape of the funnel plots seemed symmetrical for *CTLA4* C60T polymorphism, suggesting that no bias from selected studies have been included (Figure S3). The statistical results still did not show publication bias (Begg test, P = 0.32; Egger test, P = 0.18).

## Discussion

Large sample and unbiased epidemiological studies of predisposition genes polymorphisms could provide insight to etiology of diseases. This is the most comprehensive meta-analysis examined the *CTLA4* polymorphisms and the relationship to susceptibility for T1D. Its strength was based on the accumulation of published data giving greater information to detect significant differences. In total, the meta-analysis involved 58 studies for T1D which provided 30,734 cases and 40,754 controls.

Our results demonstrated that the G49A and C60T polymorphism of *CTLA4* is a risk factor for developing T1D. In the stratified analysis by ethnicity, significant associations were found in Caucasians and Middle Eastern population for the two polymorphisms in all genetic models. Significant associations were detected among East Asians for G49A polymorphism; while no associations were found for C60T polymorphism. Among Indian population, only marginal significant associations were detected for C60T polymorphism. No associations were found in Africans. The reasons that the same polymorphism plays a different role in different ethnic populations or across different studies may arise from many aspects. Firstly, T1D is a complex disease and genetic heterogeneity exists in different populations. Whole genome linkage studies on T1D have confirmed this genetic heterogeneity [66]. Secondly, clinical heterogeneity may also explain the discrepancy. Potential contribution of differences in patient populations (e.g., age and years from onset, female proportion, disease severity…) might cause different results. Thirdly, population structure difference may also contribute to the discrepancy. Different populations often have different LD patterns. The same polymorphism plays a different role in disease susceptibility in different ethnic populations, implicating that this polymorphism might not be a causal variant. The fact is that this polymorphism may be in LD with a nearby causal variant in one ethnic population but not in another. Moreover, the difference might come from type I error. Therefore, additional studies are needed to further validate ethnic difference of the effect of these polymorphisms on T1D risk Therefore, additional studies are warranted to further validate ethnic difference in the effect of these polymorphisms on T1D risk.

An important source of bias in every meta-analysis is related to the studies that have been published and thus can be included in the analysis. Nevertheless in our meta-analysis, we included many studies with negative findings. Although the funnel plot for G49A polymorphism is not symmetric, the overall results of different ethnic groups are concordant, indicating that this bias cannot affect the final result. On the other hand, funnel plot asymmetry is not always caused by publication bias. True heterogeneity may also lead to funnel plot asymmetry. For example, significant difference may be seen only in high-risk individuals, and these high-risk people are usually more likely to be included in small studies. This is particularly true in our meta-analysis because the majority of the significant associations have been observed among the studies with small sample size. Language bias or citation bias also could be an important source in this group of studies, meaning that the studies without significant findings are preferentially published in languages other than English and less likely to be cited in other articles. Finally, it is possible that an asymmetrical funnel plot arises simply by chance.

The heterogeneity of OR is high in our data, especially in the studies for African and Indian populations, based on a small number of individuals. Nevertheless, the total number of subjects included in this part of the analysis comprises the largest sample size so far. Future studies including larger numbers of Africans and Indians are necessary to clarify the consistency of findings across ethnic groups. Another possible source of heterogeneity is difference in age at onset of T1D: early or late onset. Unfortunately, it was not possible to tease out this association because the breakdown of the two types was not consistently reported.

A number of factors predict T1D, however, detailed pathogenesis mechanisms of T1D remain a matter of speculation. Polymorphisms of the *CTLA4* gene have been shown to confer susceptibility to several autoimmune diseases, due to its role in the down-regulation of the activated immune response [67]. The 49 G/G genotype of the *CTLA4* gene was associated with reduced inhibitory function of cytotoxic T-lymphocyte antigen 4 [67]. In addition, it is proposed that a reduced function of CTLA4 associated with the C allele of C60T polymorphism allows T cells to be more hyperactive and to respond to peripheral antigens to a greater degree than individuals carrying the T/T genotype, which is associated with autoimmune disease protection and increased peripheral tolerance [68], [69]. Association studies and functional data along with our meta-analysis suggest that G49A and C60T polymorphisms of *CTLA4* are risk factors for developing T1D.

Several meta-analyses addressing the same theme have been recently published [70]–[72]. However, Chen et al. and Si et al. mainly focused on the G49A polymorphism without assessing the relationship between *CTLA4* C60T polymorphism and T1D [70], [71]. Furthermore, the results reported by Tang et al. were believed to be not entirely credible for insufficient literature identification and overlapping samples [72], [73]. Compared to those previous meta-analyses, the present study has considered more studies from the literature. In addition, we also investigated the two common variants on *CTLA4* gene and genetic susceptibility to T1D. Furthermore, we also explored whether the *CTLA4* gene haplotypes were associated with T1D risk. Our results also suggest the importance of including a haplotype-based approach to assess genetic associations. Haplotype-based case–control studies are warranted to confirm our findings in the future.

In summary, this meta-analysis showed that the *CTLA4* G49A and C60T polymorphism was significantly associated with increased risk of T1D, particularly in Caucasian and Middle Eastern population. While the CTLA4 G49A and C60T polymorphisms are significantly associated with increased risk of T1D, larger cohorts of Indian and African subjects are needed to test the effect of these SNPs in these populations.

## Supporting Information

Checklist S1 (DOC)

Figure S1 **Flow chart of literature search for studies examining** ***CTLA4*** **gene polymorphism and risk of T1D.**
(TIF)

Figure S2 **Funnel plot of studies of the G49A polymorphism of** ***CTLA4*** **and T1D showing a possible excess of smaller studies with strikingly positive findings beyond the 95% CI.**
(TIF)

Figure S3 **Funnel plot for the association between** ***CTLA4*** **C60T polymorphism and T1D risk.**
(TIF)

Table S1 **Meta-analysis of haplotype combinations between G49A, and C60T polymorphisms of** ***CTLA4*** **gene and T1D risk.**
(DOCX)
