# Supplementary figures and images for: Common Variants on Cytotoxic T Lymphocyte Antigen-4 Polymorphisms Contributes to Type 1 Diabetes Susceptibility: Evidence Based on 58 Studies
Source: PLoS One. 2014 Jan 23;9(1):e85982. doi: 10.1371/journal.pone.0085982 (PMC3900458; doi:10.1371/journal.pone.0085982)

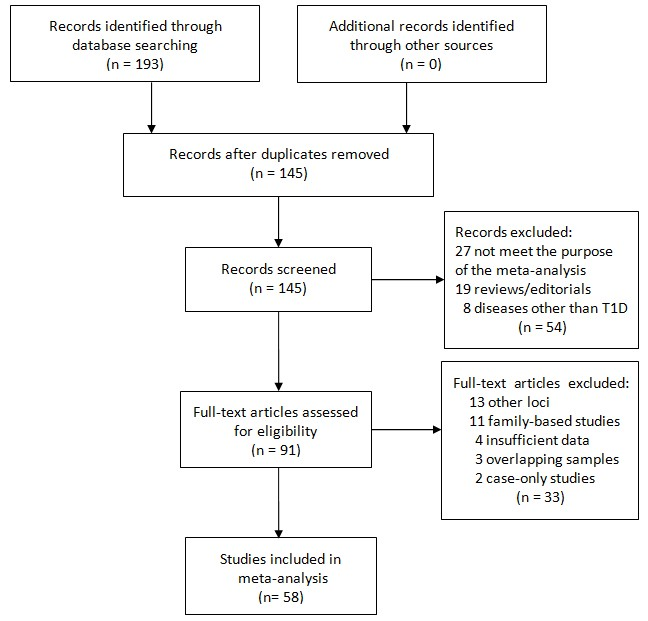

Supplement: Figure S1 — Flow chart of literature search for studies examining CTLA4 gene polymorphism and risk of T1D. (TIF) [file pone.0085982.s002.tif]

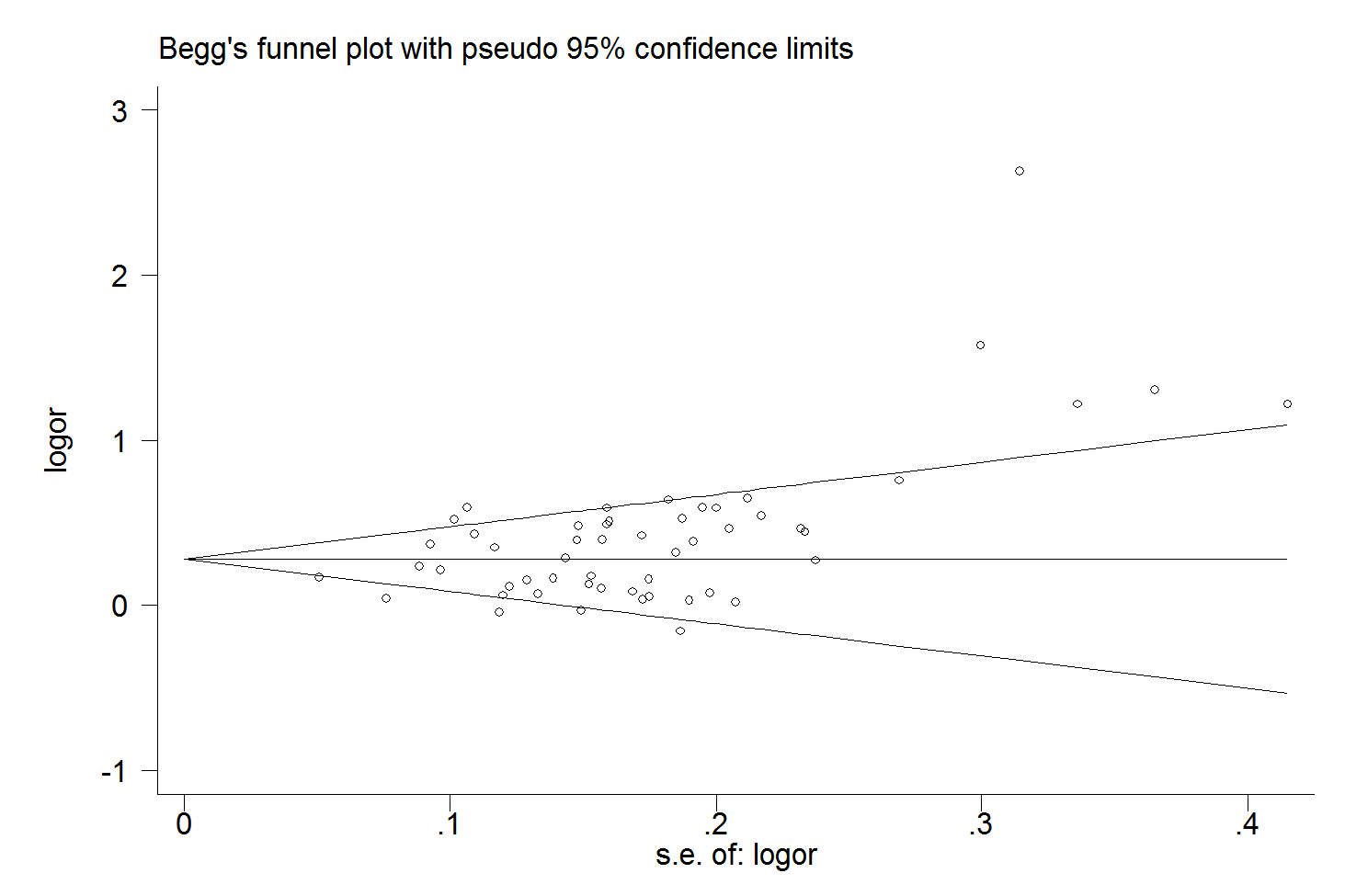

Supplement: Figure S2 — Funnel plot of studies of the G49A polymorphism of CTLA4 and T1D showing a possible excess of smaller studies with strikingly positive findings beyond the 95% CI. (TIF) [file pone.0085982.s003.tif]

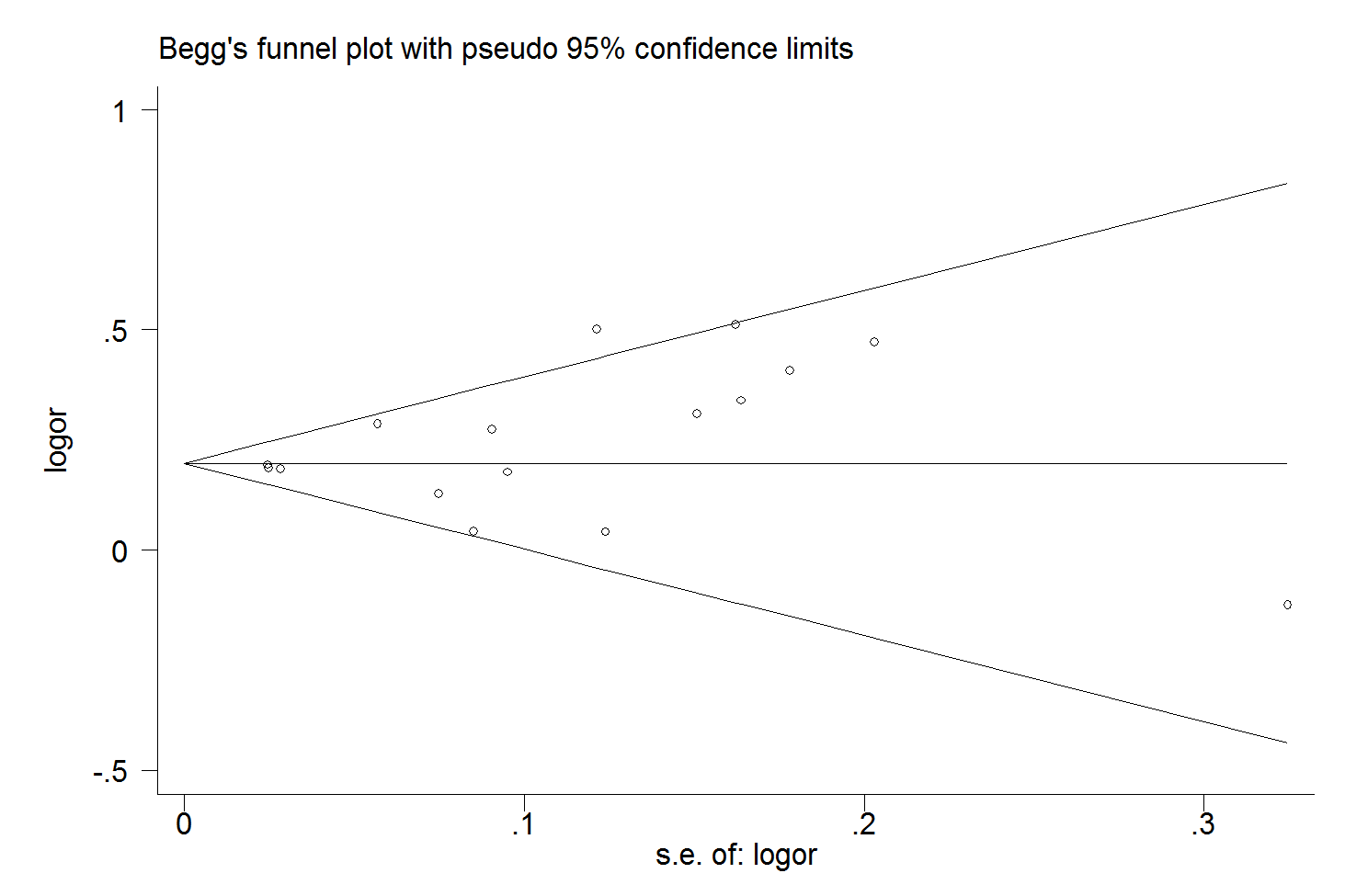

Supplement: Figure S3 — Funnel plot for the association between CTLA4 C60T polymorphism and T1D risk. (TIF) [file pone.0085982.s004.tif]
