# Supplementary material for: Common Variants on Cytotoxic T Lymphocyte Antigen-4 Polymorphisms Contributes to Type 1 Diabetes Susceptibility: Evidence Based on 58 Studies
Source: PLoS One. 2014 Jan 23;9(1):e85982. doi: 10.1371/journal.pone.0085982 (PMC3900458; doi:10.1371/journal.pone.0085982)
Supplement: Table S1 — Meta-analysis of haplotype combinations between G49A, and C60T polymorphisms of CTLA4 gene and T1D risk. (DOCX) [file pone.0085982.s005.docx]

**Table S1** Meta-analysis of haplotype combinations between G49A, and C60T polymorphisms of *CTLA4* gene and T1D risk.

| Study ^a^ | Subjects | | G-C ^b^ | | A-T ^b^ | | A-C ^b^ | |
| --- | --- | --- | --- | --- | --- | --- | --- | --- |
|  |  |  | Frequency | | Frequency | | Frequency | |
|  | Case | Control | Case | Control | Case | Control | Case | Control |
| Baniasadi [41] | 130 | 180 | 0.373 | 0.347 | 0.504 | 0.578 | 0.119 | 0.05 |
| Ikegami [43] | 1538 | 1446 | 0.624 | 0.622 | 0.227 | 0.237 | 0.135 | 0.131 |
| Douroudis (Estonia) [51] | 170 | 230 | 0.498 | 0.424 | 0.305 | 0.369 | 0.187 | 0.191 |
| Douroudis (Finland) [51] | 404 | 725 | 0.574 | 0.516 | 0.287 | 0.326 | 0.13 | 0.15 |
| Overall OR and 95% CI ^c^ | 2242 | 2581 | 1.12 (0.98-1.28) | | **0.87 (0.77-0.98)** | | 1.14 (0.80-1.63) | |

^a^ All those frequency<0.03 were ignored in the analysis.

^b^ The haplotypes are those composed in the order of G49A–C60T.

^c^ Pooled from random effect model.
